# Successful Management of Aorto-Oesophageal Fistula Following Accidental Ingestion of Chicken Bone

**DOI:** 10.7759/cureus.35959

**Published:** 2023-03-09

**Authors:** Anuj Goyal, Raja Lahiri, Nirjhar Raj Rakesh, Anshuman Darbari

**Affiliations:** 1 Surgical Gastroenterology, All India Institute of Medical Sciences Rishikesh, Rishikesh, IND; 2 Cardiothoracic & Vascular Surgery, All India Institute of Medical Sciences Rishikesh, Rishikesh, IND

**Keywords:** esophageal surgery, emergency gastroenterology and endoscopy, aortic injury, aorto-enteric fistula, esophageal foreign body

## Abstract

Accidental ingestion of foreign bodies is common in clinical practice. It is usually seen to pass through the gastrointestinal tract easily. However, in the case of impaction in the esophagus, it can lead to catastrophic conditions. Aorto-esophageal fistula is one such disastrous complication with a high fatality rate. Despite treatment, mortality rates of up to 80% have been reported in the literature, with the condition being uniformly fatal in untreated patients. We describe a case of aorto-esophageal fistula secondary to a chicken bone impaction presenting with sentinel hemorrhage and managed expeditiously and successfully with simultaneous endoscopic removal and surgical repair of the fistula.

## Introduction

Accidental foreign body ingestions are commonly seen in clinical practice. It is usually seen in children but can happen in adults accidentally or under the influence of alcohol. Most of them tend to pass through the gastrointestinal tract easily. However, a few, when impacted, can lead to complications if not removed early. Sharp objects such as pins, dentures with hooks, and fishbones have greater chances of impaction. An aorto-esophageal fistula is one such fatal complication. Most patients die before intervention due to massive hematemesis [1-3]. A few might present with an initial sentinel bleed, which should point to the diagnosis and prompt quick intervention. We describe a case of an impacted chicken bone in the esophagus, which presented late with sentinel hemorrhage and was rescued on time and managed successfully by a multidisciplinary team approach of the cardiothoracic surgery, medical and surgical gastroenterology team.

## Case presentation

A gentleman in his early forties presented to the emergency department with an alleged history of accidental ingestion of chicken bone under the influence of alcohol about a week back. He reported initial slight discomfort since the episode, which had worsened significantly in the past two days. Now he presented with two episodes of hematemesis, each containing around a cupful of blood.

**Figure 1:**
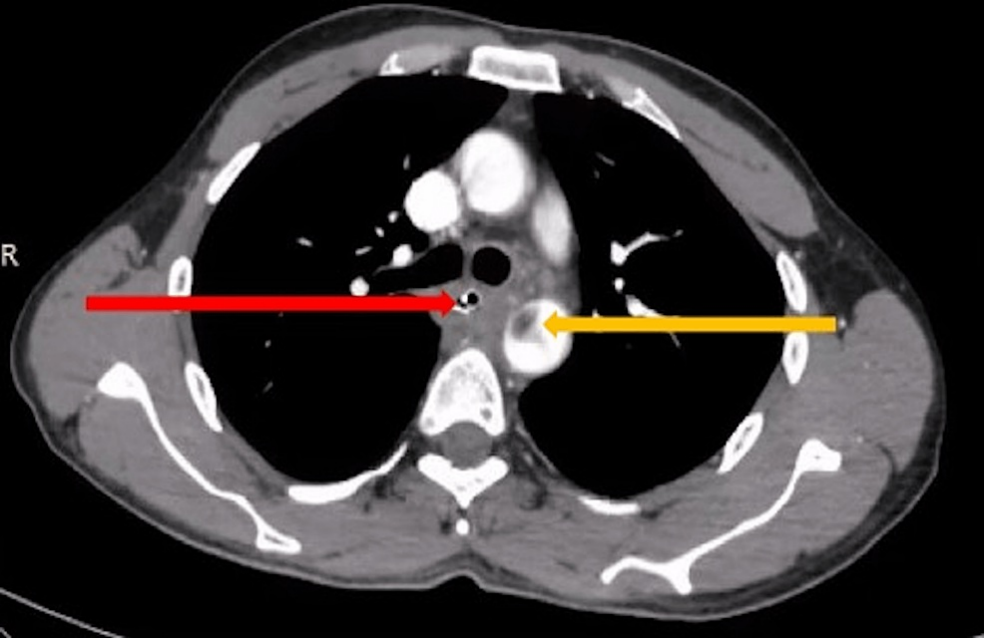
Contrast-enhanced CT thorax showing foreign body in the esophagus and thrombus in adjoining aorta The red arrow indicates a foreign body in esophagus. The yellow arrow indicates a thrombus in descending thoracic aorta.

**Figure 2:**
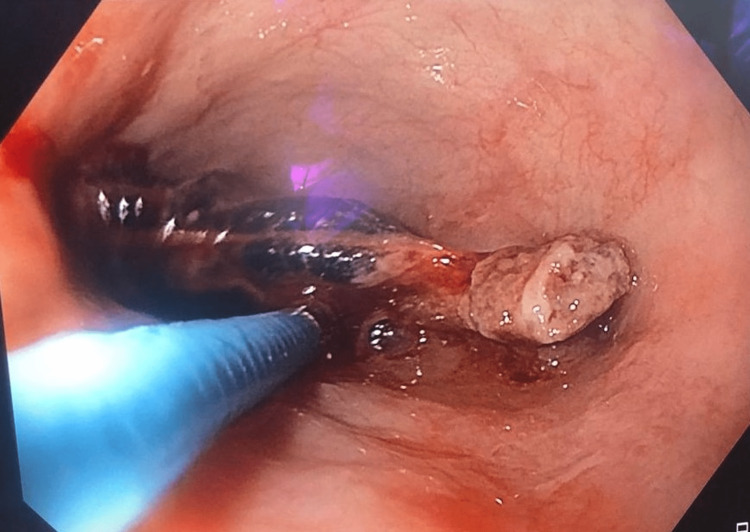
Endoscopic view of foreign body inside esophagus being held with alligator forceps

**Figure 3:**
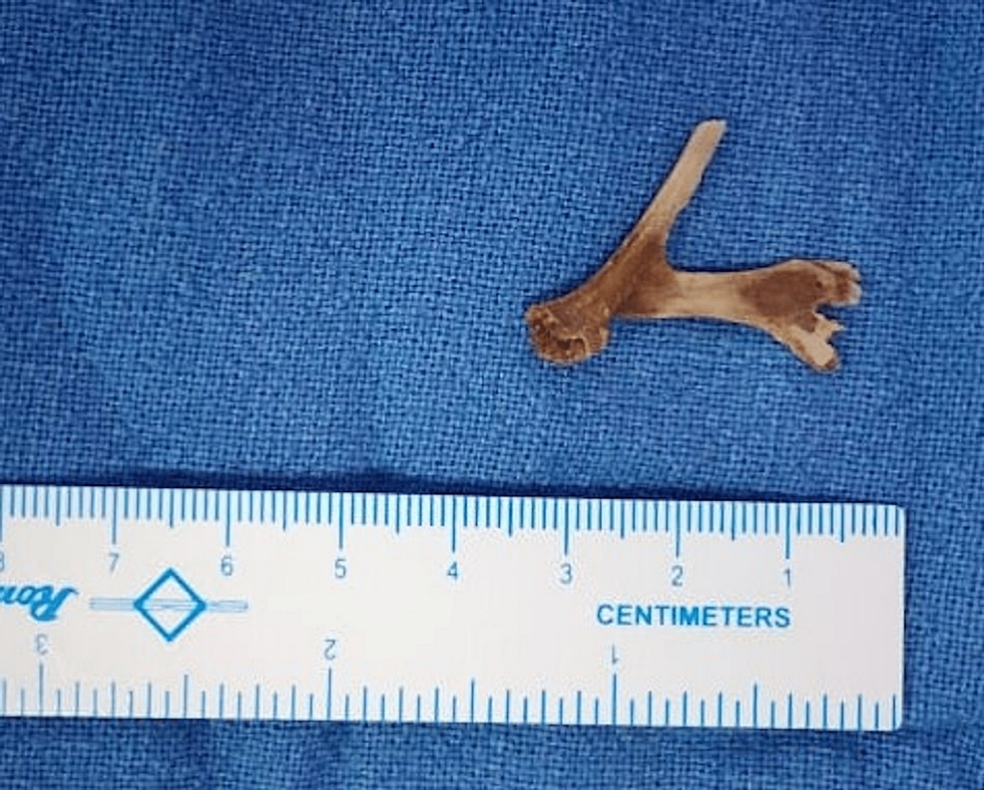
Y-shaped chicken bone after endoscopic removal

**Figure 4:**
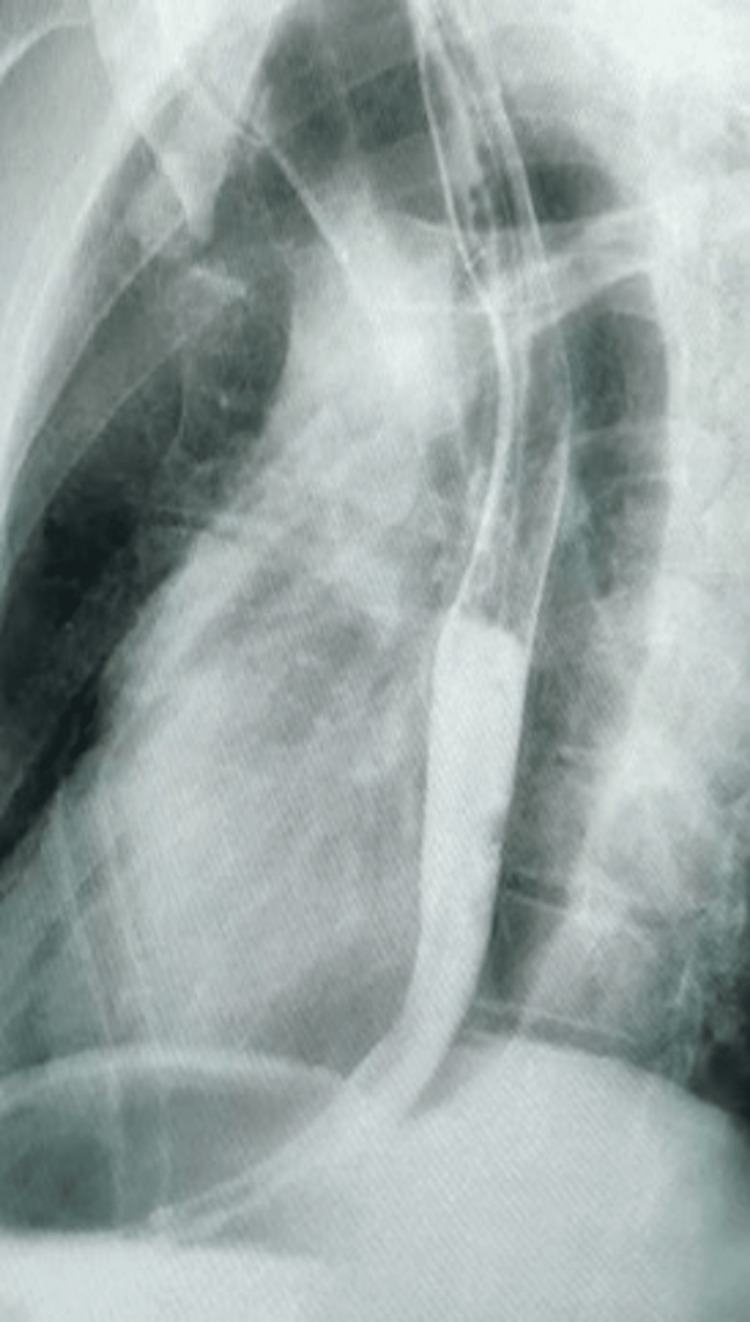
Post-operative barium swallow showing no esophageal leak

There was no history of chest pain, fever, or breathing difficulties, nor features suggestive of sepsis. Clinical examination was unremarkable except for the presence of tachycardia. In view of his presentation, he was advised intravenous contrast-enhanced computerized tomography of the thorax, which showed the presence of a suspected foreign body in the mid-thoracic esophagus, causing transmural penetration, as seen in Figure 1. A possible aortic penetration, suggested by the presence of a clot in the aorta adjoining the foreign body, was seen. A multidisciplinary team discussion involving the department of cardiothoracic surgery, medical gastroenterology and surgical gastroenterology, and a hybrid procedure was planned for the patient. The patient underwent a left posterolateral thoracotomy, with identification of the site of aortic injury and proximal and distal control of the same taken by the cardiothoracic surgeon. Intraoperative endoscopy was done, and the foreign body was removed endoscopically (Figure 2). The decision for this approach was taken due to the catapult-like shape of the foreign body, wherein transesophageal removal seemed hazardous and could potentially cause further esophageal and aortic injury during removal (Figure 3). However, after the successful removal of the foreign body, the hemostatic plug gave away, leading to torrential bleeding into the esophageal lumen. Since the aorta was already dissected and looped, it was quickly clamped, and the aorto-esophageal groove was dissected to identify the communication. The communication was divided, and the aortic defect was closed using prolene sutures reinforced with pledgets. The esophageal rent was also repaired primarily after examining the condition of the defect margin. A pleural-based flap was raised and interposed between the aorta and esophagus to prevent the development of future recurrence. The thoracotomy was closed in a standard fashion with intercostal drainage, and concomitant feeding access was achieved via a Witzel feeding jejunostomy. Post-operatively, the patient was started on enteral feeds on the first postoperative day and gradually upscaled. On the fifth postoperative day, a pleural fluid amylase was done to assess for any esophageal leak, followed by confirmation with a barium swallow. Oral sips were resumed the next day after both were confirmed to be normal. By the eighth postoperative day, the patient was started on soft solids and discharged home by the 12^th^ day (Figure 4). 

The patient was followed up after two weeks in the outpatient clinic and by telecommunication after six months. He was found to be healthy, without any dysphagia. 

## Discussion

Foreign body ingestions are common in clinical practice; however, a foreign body-induced esophageal perforation is a rare cause of an aorto-esophageal fistula. Specific guidelines for its management are difficult to formulate, and case-by-case management is the norm for all such patients. Despite treatment, mortality rates of approximately 80% have been reported in the literature, with the condition being uniformly fatal in untreated patients [1]. The classical triad associated with it includes mid-thoracic pain, an episode of sentinel bleed, followed by exsanguinating hemorrhage [4]. Various series report the incidence of the triad to be varying from 45% to 80% [5]. Other clinical features reported include backache, syncope, and shock [6]. Clinical diagnosis of the condition is difficult to attain, with the need for good quality cross-sectional imaging to confirm the same. 

Individual reports on initial management other than resuscitation have also suggested use of Sengstaken-Blakemore tubes for a potential tamponade effect on the bleed, until further definitive control of the same can be established [7,8]. Definitive treatment options for aorto-esophageal fistulae include a non-operative endovascular and endoscopic approach and a surgical approach with thoracotomy and control. Current available literature uniformly suggests using vascular stents for control of bleeding followed by further endoscopic/surgical interventions as deemed necessary [9,10]. The main issues lie in the availability of expertise and stents both, especially with regards to such patients where time is of vital importance. Stenting in areas like the aortic arch or at regions of branching is considered to be challenging due to the need for tailored stents for such locations. In addition, the cost associated with such stents becomes an important factor, especially in developing countries and for patients with a poor socio-economic background. 

Surgical access of the aorta and control of the fistula would have the advantage of controlling the bleed before any handling of the foreign body and could be considered as the ultimate fallback procedure in case of failure of an endovascular approach as well. However, surgery carries its morbidity and risk of complications. The most common access approach reported in the literature is the left lateral thoracotomy; however, the use of midline sternotomy has also been reported, albeit rarely [5]. The reasons for the same cited were to expose fistulae located on the aortic arch and also in case of need for an extra-anatomic bypass in patients needing the same [11,12]. Surgical management also allows placing an interposition flap to prevent future recurrence of the fistula.

A third approach that may be considered for such patients would be a combined approach, where the operating room would consist of a hybrid system enabling interventional radiology services within the theatre itself. This would enable immediate conversion to a surgical approach in case of failure of the endovascular approach. 

Our experience in the above case emphasizes the importance of tailoring patient care even in an emergent setting, along with maximal utilization of available resources. The aim in any such case should be to direct maximum resources towards damage control (in this case, to take control of the aortic fistula) and to do the bare minimum for other issues (esophageal rent for this patient). In the era of minimally invasive interventions, an open approach is still the most fail-safe weapon in a surgeon’s armamentarium. 

## Conclusions

Aorto-enteric fistula is a rare condition associated with a fairly common everyday scenario of esophageal foreign body impaction. Awareness regarding the possibility of the same in case of the presence of a relevant background history is very important. A sentinel hemorrhage is a warning sign and should alert the physician. Quick decision-making and prompt intervention involving multidisciplinary team care are mandatory. Possible available options for treatment must be understood by the treating team, and the involvement of the patient for the same should be done. Administrative measures to enable care to extend across multidisciplinary teams should be taken for better outcomes. Patient safety lies in identifying the problem early and managing it efficiently before a catastrophic hemorrhage occurs.
